# Supplementary material for: Tuning Hole Mobility of Individual p-Doped GaAs Nanowires by Uniaxial Tensile Stress
Source: Nano Lett. 2021 Apr 29;21(9):3894–900. doi: 10.1021/acs.nanolett.1c00353 (PMC8289290; doi:10.1021/acs.nanolett.1c00353)
Supplement: Supplementary file 1 — nl1c00353_si_001.pdf [file nl1c00353_si_001.pdf]

Supporting information

Tuning hole mobility of individual p-doped GaAs nanowires by uniaxial tensile stress

Lunjie Zeng[1*], Jonatan Holmér[1], Rohan Dhall[2], Christoph Gammer[3], Andrew M. Minor[2,4], and Eva Olsson[1*]

1 Department of Physics, Chalmers University of Technology, 412 96 Gothenburg, Sweden

2 National Center for Electron Microscopy, Molecular Foundry, Lawrence Berkeley National Laboratory, Berkeley, California 94720, United States

3 Erich Schmid Institute of Materials Science, Austrian Academy of Sciences, 8700 Leoben, Austria

4 Department of Materials Science and Engineering, University of California, Berkeley, California 94720, United States

Corresponding authors:

lunjie@chalmers.se

eva.olsson@chalmers.se

Supporting information

S1. Experiments and methods

Tensile stress was applied on individual GaAs nanowires using a Hysitron PI95 TEM holder equipped with a nanoindenter and an electrical push-to-pull (EPTP) microelectromechanical system (MEMS) device. The indentation force and the indenter displacement were measured via a transducer in the holder. When the nanowires were stressed, scanning transmission electron microscopy – nanobeam electron diffraction (STEM-NBED or 4D STEM), I-V, and monochromated electron energy loss spectroscopy (EELS) measurements were performed.

TEM

The in situ TEM experiments were performed using the TEAM 1 microscope operated at 300 kV. Further details about the TEAM1 microscope are described in [1]. A Gatan K2 direct electron detector camera attached to the TEAM 1 microscope was used to record the NBED maps. In the STEM-NBED measurements, the electron beam was focused to around 2 nm in diameter with a convergence semi-angle of ∼2 mrad. A "bullseye" patterned condenser aperture was used to improve the precision of strain measurements using STEM-NBED [2]. The electron beam was scanned over an area with the size of 2 µm × 140 nm with a step size of about 4 nm. At each beam position, a NBED pattern was acquired with an exposure time of 2.5 ms using the K2 camera. Each diffraction pattern was obtained with the full frame of the camera, giving rise to 1920 × 1792 pixels in each diffraction pattern. The lattice distances and, hence, the lattice strains were measured directly and accurately based on the distances between the Bragg spots in NBED patterns [3]. For the EELS measurement, the Wien-type monochromator in TEAM1 microscope was used. A Gatan image filter (GIF) Tridium was used for collecting EELS spectra. Beam convergence semi-angle was set to ~ 20 mrad and the collection angle for EELS was ~ 25 mrad. The energy resolution of the setup was estimated to

be ~ 110 meV based on the full-width-at-half-maximum (FWHM) of the zero loss peak. STEM-EELS spectrum images were acquired from the same nanowire area during in situ tensile test. The exposure time for each spectrum was set at 50 ms. No changes in crystal structure and electrical transport properties of the nanowire due to the illumination of the electron beam in TEM were observed.

EPTP device

The EPTP device consists of a movable part and a fixed part, which are connected by springs. The nanowires were mounted between the movable and fixed parts on the MEMS device. The nanoindenter in the holder pushed the device and effectively applied a tensile force on the nanowire along the nanowire length direction through the push-to-pull mechanism. The indentation force and the indenter displacement were measured via a transducer in the holder. The nanowires were also connected to the electrodes on the MEMS device. The electrical transport properties measurements were performed by sweeping the voltage applied on the nanowire from −4 to 4 V and measuring the current going through the nanowire. The electric current limit in the *I*–*V* measurements was set to 1 μA. During the electrical measurements, the electron beam was blanked.

Nanowire growth

The GaAs nanowires used in this study were grown on Si(111) substrate using a molecular beam epitaxy (MBE) system by a self-catalysed vapor-liquid-solid (VLS) method. The substrate temperature was kept at 630°C during growth. An As_4 flux of 11×10^{-6} Torr was kept for 45 min for the nanowire growth. The p-doping was achieved by adding a flux of beryllium during

growth of the nanowires. The nominal carrier concentration is ≈3.5×10^{19} cm^{-3} estimated from 4-probe electrical conductivity measurements [4].

Nanowire transfer

For the in situ TEM studies, individual nanowires were lifted out and mounted on the EPTP devices in a FEI Strata focus ion beam – scanning electron microscope (FIB-SEM) instrument. The nanowires were put across the gap with a width of about 2 μm between movable and fixed part of the EPTP device. This gap allows for the TEM investigation of the nanowires. The nanowire was connected to the electrical contacts on the EPTP device with electron beam induced deposition (EBID) and ion beam induced deposition (IBID) of Pt in FIB-SEM [5]. After nanowire transfer, the EPTP device was mounted on the Hysitron PI95 nanoindenter TEM holder for in situ TEM investigations.

Supporting information

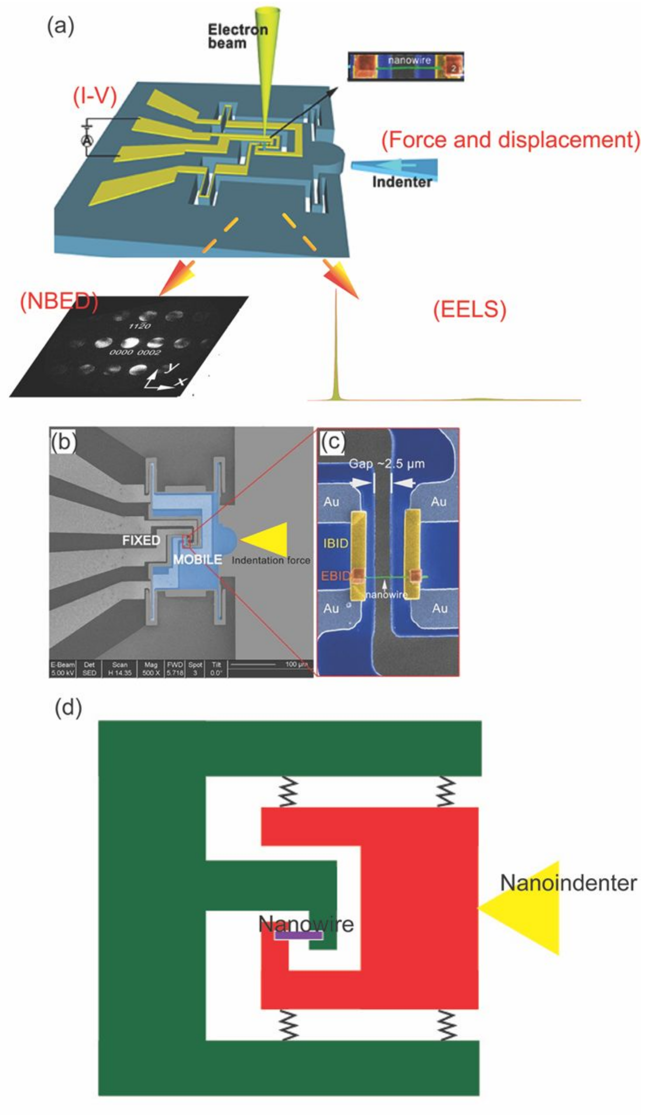

Figure S1. (a) A schematic of the EPTP MEMS device and the in situ TEM experimental setup. The MEMS device was fixed on a nanoindentation TEM holder with electrical measurement capability. There are electrical contacts (in yellow) on the top surface of the device. Individual nanowires were mounted across a gap between the movable and fixed parts of the MEMS device. The nanoindenter in the TEM holder was used to push the movable part of the MEMS device, effectively applying tensile stress on the nanowire.

(b) A scanning electron microscopy (SEM) image of the sample area of the EPTP MEMS device. The movable part is marked in blue. The yellow arrow shows the direction of the indentation force from the nanoindenter.

(c) A magnified image of the area marked by the red window in (b), showing the Au electrodes on the MEMS device, ion beam induced deposition (IBID) of Pt and electron beam induced deposition (EBID) of Pt by focus ion beam – scanning electron microscope (FIB-SEM). EBID of Pt was used to fix nanowires on the MEMS device across the gap between the movable and fixed parts of the device.

(d) A schematic of the push-to-pull mechanism. The nanoindenter pushes the movable (drawn in red) part in the EPTP MEMS device. The green part is stationary, so a tensile force is applied on the nanowire in effect.

S2. Crystal structure of the GaAs nanowires

The GaAs nanowires used in this study have Zinc Blende structure. The nanowires have similar dimensions. The growth direction of the nanowires is [111]. There are twin domains in the nanowires. The twin plane is the (111) plane. When the electron beam is aligned with the [1-10] zone axis, twin domains are visible both in the atomic resolution STEM ADF images and in selected area electron diffraction (SAED) patterns. When the electron beam is aligned with [2-1-1] direction, the twin structure is not visible.

We note that the twin structure in the nanowires do not change during in situ straining, so the twin structure should not contribute to the variation in conductivity of the nanowires.

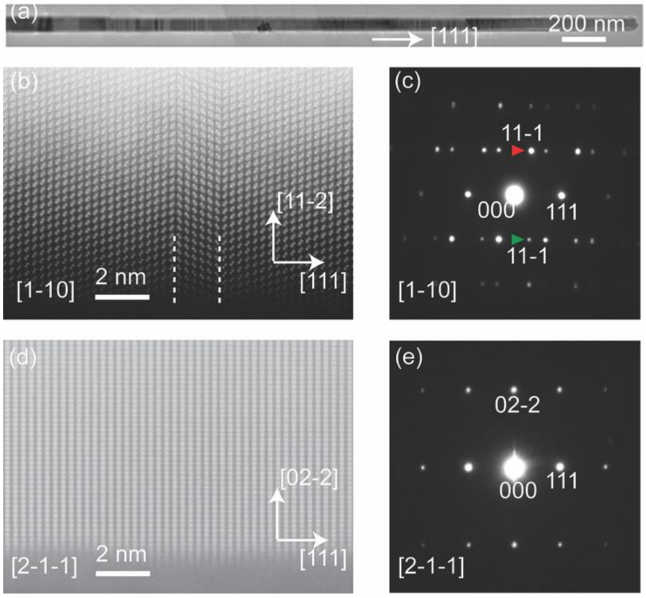

Figure S2. (a) A low magnification TEM bright field image showing the overview of a GaAs nanowires.

(b) An atomic resolution STEM ADF image with the electron beam aligned along the [1-10] zone axis of the nanowires. Twin structure is visible in the image. Two twin boundaries are

marked with dashed lines. The nanowire growth direction is [111]. The twin boundaries are perpendicular to the [111] direction. In other words, the twin plane is the (111) plane.

(c) A SAED pattern of the nanowire with the electron beam aligned with the [1-10] zone axis. Diffraction spots from 2 twin domains are visible. The (11-1) diffraction spots (green and red arrows) for the 2 twin domains are indexed. The twin domains share the {111} diffraction spots in the SAED pattern.

(d) An atomic resolution STEM ADF image with the electron beam aligned with the [2-1-1] zone axis of the nanowire. Along this zone axis, twin structure is not visible.

(e) A SAED pattern of the nanowire with the electron beam aligned with the [2-1-1] zone axis. Extra diffraction spots from twin domains are absent in the pattern.

S3. NBED pattern

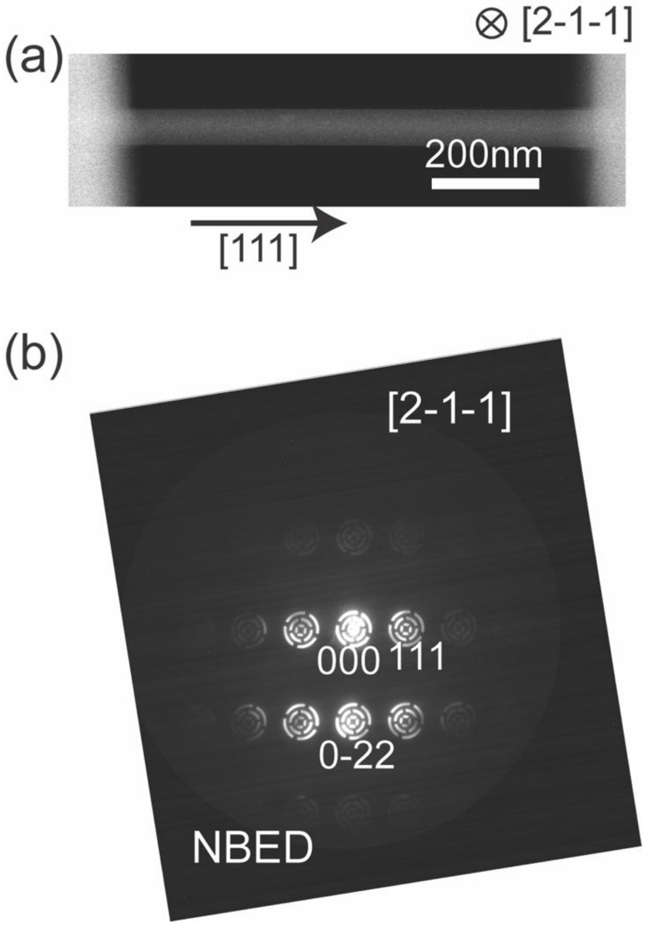

Figure S3. (a) A STEM ADF image of a GaAs nanowire. The nanowire growth (length) direction is the [111] direction. The image is taken with the electron beam direction close to the [2-1-1] zone axis.

(b) A typical NBED pattern from STEM-NBED mapping over the nanowire. The NBED patterns for STEM-NBED mapping were acquired with the electron beam direction close to the [2-1-1] zone axis. The “bullseye” pattern within the center and diffraction disks is from the “bullseye” patterned condenser aperture used in the STEM-NBED measurements.

S4. Uncertainty in the STEM-NBED strain measurement

The uncertainty or error of the STEM-NBED strain measurement was evaluated by analyzing variations in measured strain values in a nanowire area where there is considered to be no lattice distortion.

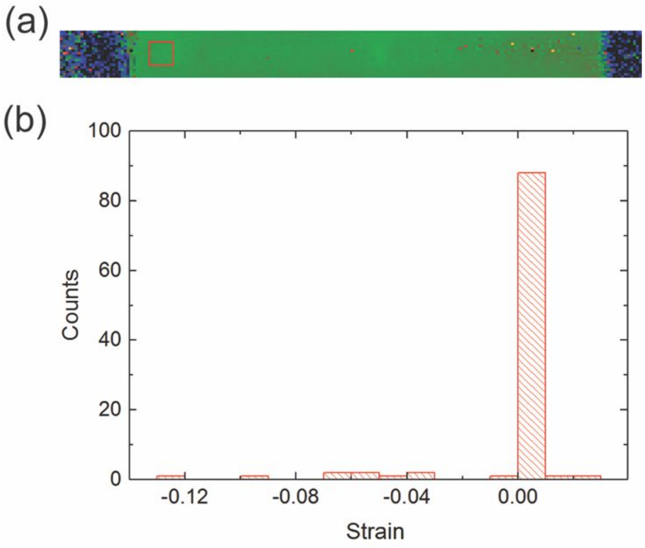

Figure S4. (a) Strain map of the nanowire without applied stress obtained using STEM-NBED. The same strain map is shown in Figure 3 in the main text. The fluctuation of strain values in the area outlined by the red window is used for assessing the uncertainty in strain measurement.

(b) Strain distribution within the area marked by the red window in (a). Since there is no applied strain within this area, the distribution of the strain values is an indication of the uncertainty in our strain measurement. The standard deviation of strain in the area and thus the uncertainty in the strain measurement is calculated to be ~ 0.06%.

S5. Extracting electrical transport property parameters in a nanoscale metal-semiconductor-metal system by quantitatively analyzing I-V characteristics

In the in situ TEM measurements, single p-GaAs nanowires are sandwiched between two metallic contacts, forming a metal-semiconductor-metal (M-S-M) system. At the M-S interfaces, Schottky barriers are established. The nanowire with resistance R_{NW} can be considered as in series with the two Schottky barriers. The I-V characteristics of the M-S-M system are thus determined by the nanowire properties, such as R_{NW} and doping concentration, and the Schottky barrier parameters, including barrier height (ϕ_1 and ϕ_2), size of the contact area (S_1 and S_2) and shunt resistance (R_{sh1} and R_{sh2}) [6,7].

When a bias V_{total} is applied on the M-S-M system, it is distributed across the Schottky barriers and the nanowire. As a result,

$$V_{total} = V_1 + V_{NW} + V_2 \,. \tag{1}$$

When under bias, one of the barriers is reverse-biased and the other one is forward-biased. The I-V relation for the forward-biased Schottky barrier is usually described by the thermionic emission theory:

$$I_f = SA^*T^2 exp\left(-\frac{q\phi}{kT}\right)\left[exp\left(\frac{qV}{nkT}\right) - 1\right] + \frac{V}{R_{sh}}\,, \tag{2}$$

Where I_f is the current going through the Schottky barrier, S is the size of the M-S junction area, $A^* = 4\pi m^* qk^2/h^3$ is the Richardson constant of the semiconductor, k is the Boltzman constant, q is the magnitude of electronic charge, h is Plank's constant, m^* is the hole effective mass in GaAs ($0.45m_0$, where m_0 is the electron rest mass), T is the absolute temperature, ϕ is the Schottky barrier height, V is the voltage drop across the forward-biased

barrier, and R_{sh} is the shunt resistance of the barrier. n is the ideality factor of the barrier. It describes the deviation of the barrier from an ideal Schottky barrier. For an ideal Schottky barrier, $n = 1$. The deviation of n from 1 originates from the detailed interface structure at the M-S contact.

For the reverse-biased Schottky barrier, thermionic field emission theory is often found to better describe the current-voltage characteristics than the thermionic emission model [6]. According to the thermionic field emission theory,

$$I_r = -I_0 exp\left[V\left(\frac{q}{kT} - \frac{1}{E_0}\right)\right] + \frac{V}{R_{sh}}, \tag{3}$$

with

$$I_0 = \frac{SA^*T(\pi qE_{00})^{\frac{1}{2}}}{k} exp\left(-\frac{\phi}{qE_0}\right)\left[q(V-\chi) + \frac{\phi}{\cosh^2(\frac{qE_{00}}{kT})}\right]^{\frac{1}{2}}, \tag{4}$$

where χ is the distance between the Fermi energy (E_F) and the top of the valence band in the nanowire, and

$$E_0 = E_{00}\coth\left(\frac{qE_{00}}{kT}\right), \tag{5}$$

$$E_{00} = \frac{\hbar}{2}\left[\frac{N_d}{m^*\varepsilon_s\varepsilon_0}\right]^{\frac{1}{2}}, \tag{6}$$

where N_d is the doping concentration at the M/S interface, m^* is the hole effective mass, ε_s is the relative permittivity of the nanowire (it is set to the same value as that of bulk GaAs, 12.9), and ε_0 is the permittivity of free space.

Assuming Barrier 2 is reverse-biased and Barrier 1 is forward-biased and because the current going through the Schottky barriers and the nanowire should be the same, we have

$$I = I_f(V_1, R_{sh1}) = I_r(V_2, R_{sh2}) = \frac{V_{NW}}{R_{NW}}. \tag{7}$$

Experimental I-V curves were fitted to the model above. Electrical parameters of the nanowire and the Schottky barriers were extracted. Those parameters include the nanowire conductance, the Schottky barrier height, the size of the barrier area. It is worth noting that the high bias region of the I-V characteristics is dominated by the nanowire conductance or resistance, while the low bias region is governed by the Schottky barriers, especially the reverse-biased Schottky barrier. This property and the quantitative analysis of the I-V curves allow the reliable extraction of R_{NW} values by decoupling the effect of the electrical contacts. In addition, according to the model shown above, a slight difference in barrier height and barrier area size between the two Schottky barriers can result in asymmetry in I-V characteristics, which were observed in some nanowires (e.g., Figure S8 and S9).

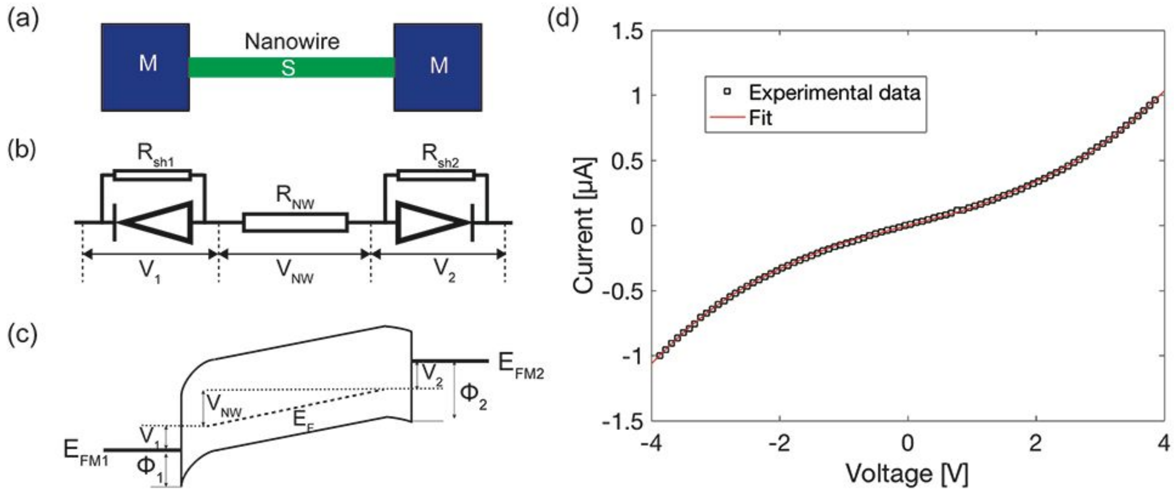

Figure S5. (a) A schematic showing that the p-type GaAs nanowire is connected to two metal contacts in the in situ TEM setup. They form a metal-semiconductor-metal (M-S-M) system. (b) A diagram of the equivalent circuit of the M-S-M system. The GaAs nanowire is considered as a resistor sandwiched between and in series with two head-to-head Schottky barriers that are formed between the nanowire and the metal contacts. V_1, V_{NW}, and V_2 are the voltage drops across the Schottky barrier to the left (barrier 1), the nanowire, and the Schottky barrier

to the right (barrier 2), respectively, when a bias is applied. R_{NW} is the resistance of the nanowire. R_{sh1} and R_{sh2} denote the shunt resistances of the two Schottky barriers.

(c) A band diagram of the M-S-M system under bias. E_{FM1} and E_{FM2} are Fermi levels of the two metal contacts. The Fermi level in the nanowire is denoted by the dashed line and E_F. ϕ_1 and ϕ_2 are the barrier heights of the two Schottky barriers. The voltage drops V_1, V_{NW}, and V_2 are also shown in the diagram.

(d) An example of the experimental I-V characteristics. Result of data fitting based on the M-S-M model is also shown.

S6. Parameters extracted by fitting the I-V curves to the theoretical model

Table I shows a summary of the main parameters (except R_{NW}) that are used in the theoretical model to describe the I-V characteristics of the 3 nanowires used. Fitting results of R_{NW} are shown in Figure 3 (Nanowire 1), S8 (Nanowire 2), and S9 (Nanowire 3). n is the ideality factor of the Schottky barriers, χ is the distance between the Fermi level and the top of the valence band, S is the size of the M-S contact area, N_d is the doping concentration, ϕ is the Schottky barrier height, and R_{sh} is the shunt resistance. The subscripts of the parameters correspond to the two M-S contacts. n, χ and S do not change with strain and are set to be the same for the 3 nanowires. We note that n has little effect on the data fitting. χ is deduced from nominal doping concentration in the nanowires, which is ~ $3.5e^{25}m^{-3}$. S is determined from SEM imaging. N_d, ϕ, and R_{sh} are extracted from data fitting. Dopant concentration (N_d) may be inhomogeneous along the nanowires, so it is reasonable that N_{d1} and N_{d2} may be different. Dopant concentration can also be different between nanowires due to the uneven incorporation of the dopant atoms between the nanowires during nanowire growth. The parameters that may vary due to strain are Schottky barrier height and shunt resistance. More details of ϕ and R_{sh} for the 3 nanowires are shown in S7, S8, and S9.

Supporting information

Table I. Main parameters used for and obtained from fitting the I-V curves to the theoretical model.

	n_1	n_2	χ [V]	S_1 [m^2]	S_2 [m^2]	N_{d1} [m^{-3}]	N_{d2} [m^{-3}]	ϕ_1 [eV]	ϕ_2 [eV]	R_{sh1} [Ω]	R_{sh2} [Ω]
Nanowire 1	2	2	0.02	6e-12	6e-12	4.65e23	4.65e23	0.581-0.594	0.571-0.582	3.0-3.3e6	2.9-3.2e6
Nanowire 2	2	2	0.02	6e-12	6e-12	2.5e24	9e23	0.56	0.49	1e10	1e10
Nanowire 3	2	2	0.02	6e-12	6e-12	2e24	9e23	0.52	0.494	1e10	1e10

S7. Changes in Schottky barrier height (ϕ) and shunt resistance (R_{sh}) in Nanowire 1 due to applied stress.

Schottky barrier height (ϕ) and shunt resistance (R_{sh}) of the two electric contacts were extracted by fitting the I-V characteristics to the theoretical model shown in S5. Schottky barrier height of a M-S contact normally depends on the band gap and doping level of the semiconductor. The insulating native oxide layer on the surface of the nanowire can also contribute to ϕ. A shunt resistance is an effective resistance in parallel with the barrier. Due to surface depletion or surface charge accumulation, there may be surface charges on the nanowire surface. These surface charges can form an additional conducting channel at the M-S contact, which is the origin of the shunt resistance. For an ideal Schottky barrier, $R_{sh} = \infty$. For a nanowire, R_{sh} is normally ~ $G\Omega$. In Nanowire 1, R_{sh} is a few $M\Omega$. The relatively low R_{sh} may be due to an accidently modified surface structure in Nanowire 1 during the deposition of the contacts in FIB-SEM. The effect of R_{sh} is important at low bias regime, but it has minor effect on the extracted R_{NW} values, which determines the I-V behavior of the nanowire at high bias regime.

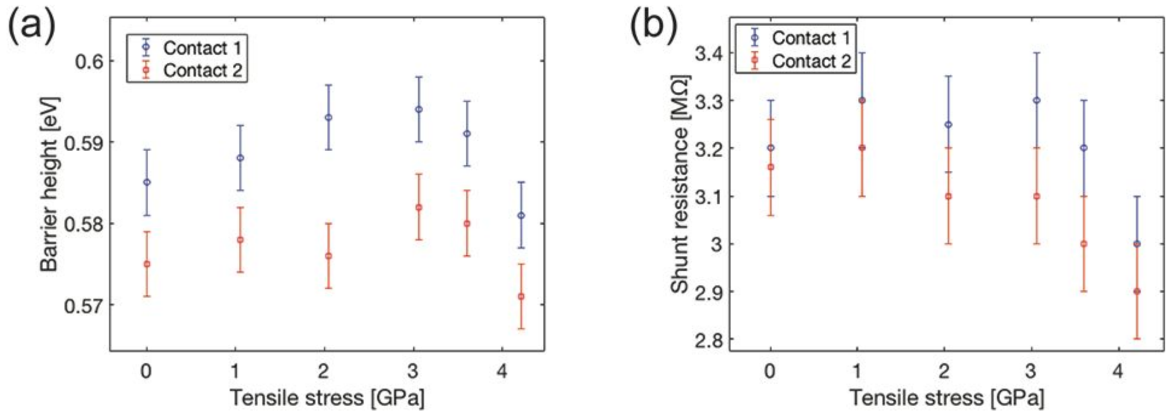

Figure S7. (a) Schottky barrier heights in Nanowire 1 as a function of applied stress.

(b) Shunt resistances in Nanowire 1 as a function of applied stress.

S8. Repeated straining and I-V measurements on Nanowire 2

In Nanowire 2, the I-V curves are slightly asymmetric on the positive and negative bias regions. This is attributed to a slight difference between the two Schottky barriers. However, the conductance of the nanowire can still be reliably extracted by using the theoretical model as shown in Figure S5. The same trend in the change of I-V curves in the high bias region as that shown in the main text is observed. The measurements were repeated. The changes in R_{NW} and ϕ as a function of applied stress were also determined by fitting the I-V curves to the theoretical model shown in S5. Shunt resistances of the two contacts are both 10 $G\Omega$ (Table I) and do not change with applied stress.

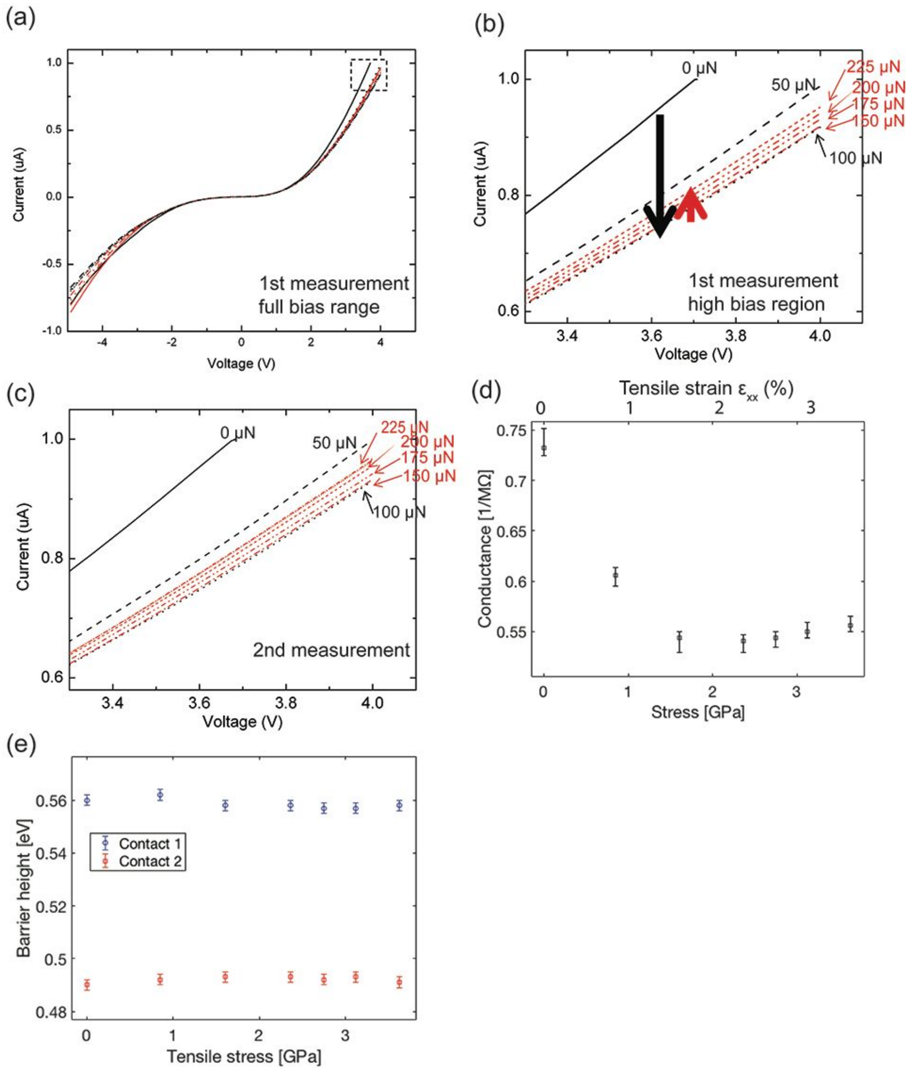

Figure S8. (a) I-V measurement on a p-GaAs nanowire (Nanowire 2) with the applied bias in the range from ~-4V to 4V. The I-V curves in the marked window in the upper right corner is magnified and shown in (b).

(b) I-V curves from the in situ measurement and in the bias range from ~3.3V to 4V (high bias and current range). The applied indentation force is indicated for each I-V curve. The curves in black show a decrease in slope and total current with increasing force, which is also indicated by the thick black arrow. The red curves show that the slope of the I-V curves starts to gradually increase when the applied indentation force is above 100 μN. The increase in current and I-V slope is indicated by a thick red arrow.

(c) Repeated I-V measurement result on the same nanowire as in (a) and (b).

(d) The conductance of the nanowire as a function of tensile stress and strain. The initial decrease in conductance at around 1.5 GPa is about 25%. The conductance of the nanowire at around 4 GPa stress is around 80% of the original value.

(e) The Schottky barrier heights (ϕ) of the two electrical contacts in the nanowire. Note that the two barriers have different barrier heights. The barrier heights show insignificant change during in situ straining.

S9. In situ straining and I-V measurements on Nanowire 3 using a scanning tunneling microscope – scanning electron microscope (STM-SEM) setup

A STM-SEM setup was also used to study the effect of uniaxial tensile strain on the I-V characteristics of the GaAs nanowires. A more detailed description of the setup can be found in a previous study [8]. The as-grown nanowires on p-doped Si substrate were directly measured. The tip of single nanowires was welded onto the STM probe using Pt deposition in a FIB-SEM, while the bottom of the nanowires was connected to the growth substrate. By moving the STM probe along the nanowire length direction, tensile strain was applied on the nanowires. The magnitude of tensile strain was determined by measuring the elongation of the nanowires based on SEM images. In the example shown here, I-V curves from 2 subsequent measurements performed on the same nanowire are presented. The nanowire was first gradually strained up to ~ 3%. Then the STM probe was moved back to its original position, as closely as possible. However, the nanowire was slightly elongated compared to its original length, at the beginning of the second measurement. Thus, in the second measurement, the nanowire was stained by an amount from ~ 0.4% to ~ 4%. The conductance of the nanowire was extracted using a similar model as that shown in Figure S5. In the STM-SEM setup, we have a semiconductor-semiconductor-metal (S-S-M) configuration instead of the M-S-M system in the in situ TEM setup. However, there is also an energy barrier at the S-S interface between the nanowire and the substrate due to the difference in band structure between GaAs and Si. Thus, a similar model as for the case of M-S-M can be used to model the I-V characteristics that are obtained from the STM-SEM measurements. Moreover, the I-V characteristics in the high bias region (linear region) are mostly determined by the nanowire conductance, while the energy barriers at the two electrical contacts predominantly affect the I-V relationship at relatively low bias region (nonlinear region). As a result, the change in

the parameters of the contacts in the model has insignificant effect on the extraction of the nanowire conductance from the I-V curves. The obtained conductance change as a function of strain shows the same trend as that shown in other examples, i.e. there is a decrease in conductance at relatively lower stain levels, followed by a gradually increase at higher strain levels. The changes in R_{NW} and ϕ as a function of applied stress were also determined by fitting the I-V curves to the theoretical model shown in S5. Shunt resistances of the two contacts are both 10 $G\Omega$ (Table I) and do not change with applied stress.

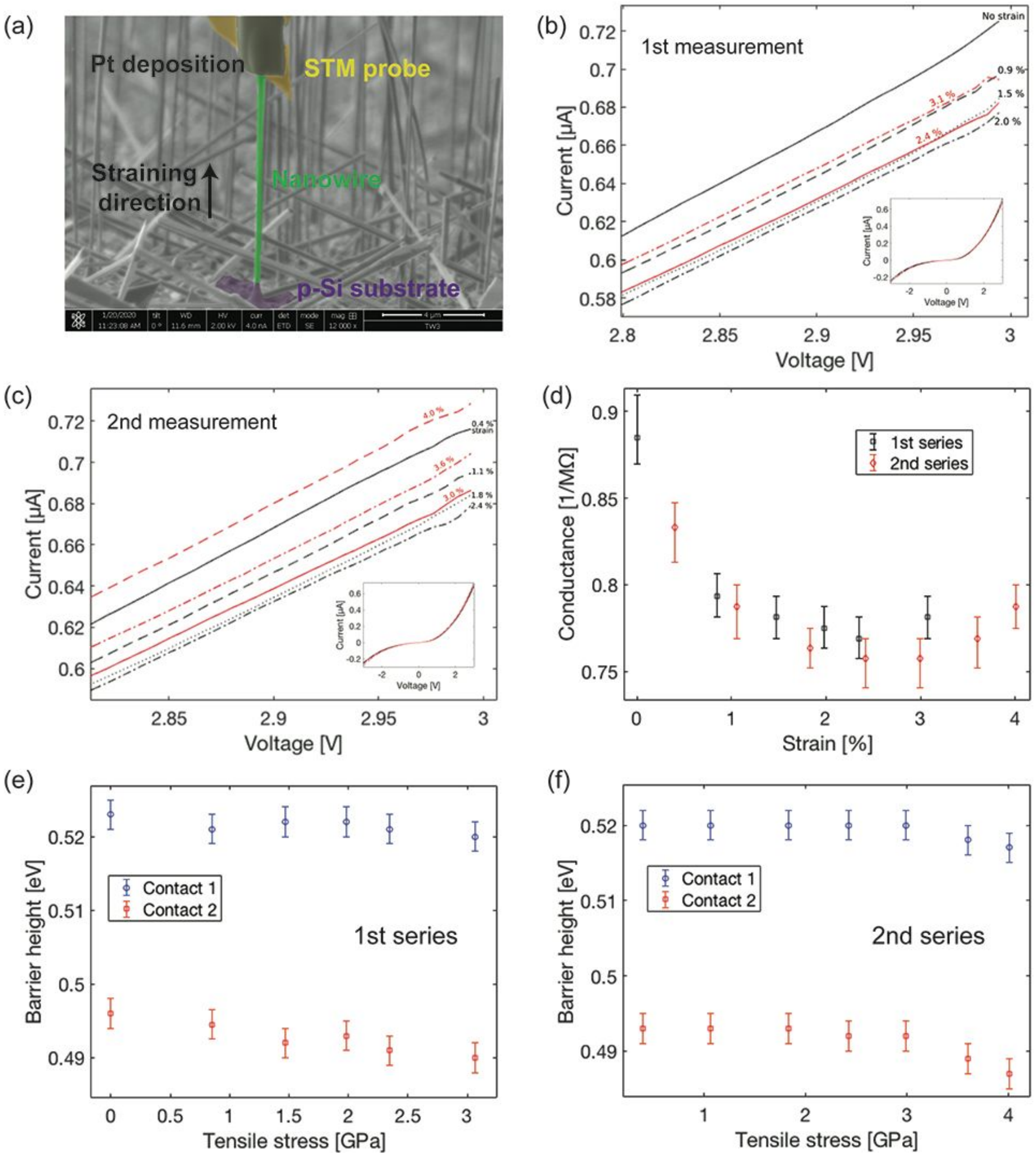

Figure S9. (a) A SEM picture showing the in situ STM-SEM experimental setup. The main components in the setup, (i.e. the Au STM probe, the nanowire, the nanowire growth substrate, and the deposited Pt for welding the nanowire on the STM probe) are false-colored. The straining direction for the in situ measurements is also indicated. Note that only part of the substrate close to the nanowire bottom is colored. Surrounding nanowires grown on the same substrate are also visible in the SEM image. Care was taken to avoid contacting other nanowires while performing in situ straining measurements.

(b) High bias region of the I-V curves from the 1st measurement. The I-V curves in a larger bias range are shown as the inset. The strain value corresponding to each I-V curve is also shown. In the high bias region, the electrical current decreases with increasing strain at low strain levels (curves shown in black). When the strain is larger than ~2%, the current starts to increase with increasing strain (curves shown in red).

(c) High bias region of the I-V curves from the 2nd measurement. The I-V curves in a larger bias range are shown as the inset. Similar as in (b), the curves in black show the decrease in current for an applied bias at low strain levels, while the curves in red show that the current increases with strain at large strain levels.

(d) Conductance of the nanowire as a function of tensile strain for the two measurement series shown in (b) and (c).

(e) Schottky barrier heights at the two contacts as a function of applied stress extracted from I-V curves in the 1st measurement series. There is insignificant change in barrier heights during in situ straining.

(f) Schottky barrier heights at the two contacts as a function of applied stress extracted from I-V curves in the 2nd measurement series. There is insignificant change in barrier heights during in situ straining.

S10. In situ monochromated valence EELS of the GaAs nanowire under tensile stress

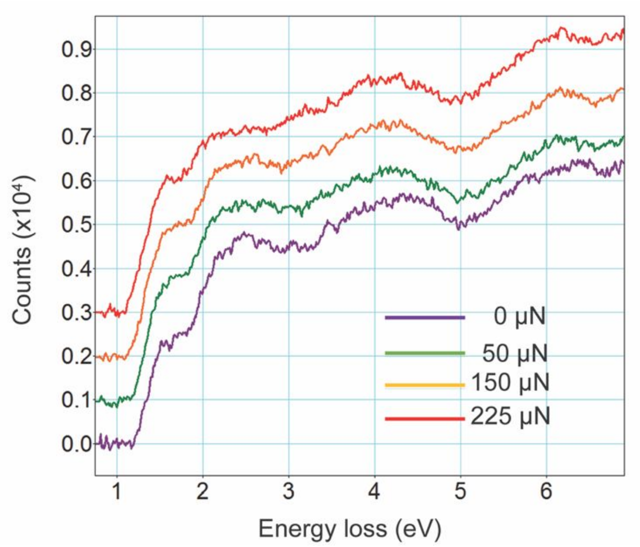

Figure S10. Monochromated valence EEL spectra of the GaAs nanowire under tensile stress. The spectra are shifted along the vertical axis for clarity. In each spectrum, there is an onset in EELS signal around 1.4 eV, which is corresponding to the band gap of GaAs. The features between 2 and 7 eV in the spectra are signals from other interband transitions. Tensile stress shifts the band gap onset to lower energy, rather than changing other main features in the spectrum that originate from interband transitions.

Supporting information

References:

(1) Dahmen, U.; Erni, R.; Radmilovic, V.; Ksielowski, C.; Rossell, M.-D.; Denes, P. Background, Status and Future of the Transmission Electron Aberration-Corrected Microscope Project. *Philos. Trans. R. Soc. A Math. Phys. Eng. Sci.* **2009**, *367* (1903), 3795–3808. https://doi.org/10.1098/rsta.2009.0094.

(2) Zeltmann, S. E.; Müller, A.; Bustillo, K. C.; Savitzky, B.; Hughes, L.; Minor, A. M.; Ophus, C. Patterned Probes for High Precision 4D-STEM Bragg Measurements. *Ultramicroscopy* **2020**, *209*, 112890. https://doi.org/10.1016/j.ultramic.2019.112890.

(3) Ozdol, V. B.; Gammer, C.; Jin, X. G.; Ercius, P.; Ophus, C.; Ciston, J.; Minor, A. M. Strain Mapping at Nanometer Resolution Using Advanced Nano-Beam Electron Diffraction. *Appl. Phys. Lett.* **2015**, *106* (25), 253107. https://doi.org/10.1063/1.4922994.

(4) Casadei, A.; Krogstrup, P.; Heiss, M.; Röhr, J. A.; Colombo, C.; Ruelle, T.; Upadhyay, S.; Sørensen, C. B.; Nygård, J.; Fontcuberta i Morral, A. Doping Incorporation Paths in Catalyst-Free Be-Doped GaAs Nanowires. *Appl. Phys. Lett.* **2013**, *102* (1), 013117. https://doi.org/10.1063/1.4772020.

(5) Zeng, L.; Gammer, C.; Ozdol, B.; Nordqvist, T.; Nygård, J.; Krogstrup, P.; Minor, A. M.; Jäger, W.; Olsson, E. Correlation between Electrical Transport and Nanoscale Strain in InAs/In 0.6 Ga 0.4 As Core–Shell Nanowires. *Nano Lett.* **2018**, *18* (8), 4949–4956. https://doi.org/10.1021/acs.nanolett.8b01782.

(6) Zhang, Z.; Yao, K.; Liu, Y.; Jin, C.; Liang, X.; Chen, Q.; Peng, L. M. Quantitative Analysis of Current-Voltage Characteristics of Semiconducting Nanowires: Decoupling of Contact Effects. *Adv. Funct. Mater.* **2007**, *17* (14), 2478–2489. https://doi.org/10.1002/adfm.200600475.

(7) Elhadidy, H.; Sikula, J.; Franc, J. Symmetrical Current-Voltage Characteristic of a

Metal-Semiconductor-Metal Structure of Schottky Contacts and Parameter Retrieval of a CdTe Structure. *Semicond. Sci. Technol.* **2012**, *27* (1), 015006. https://doi.org/10.1088/0268-1242/27/1/015006.

(8) Holmér, J.; Zeng, L.; Kanne, T.; Krogstrup, P.; Nygård, J.; de Knoop, L.; Olsson, E. An STM – SEM Setup for Characterizing Photon and Electron Induced Effects in Single Photovoltaic Nanowires. *Nano Energy* **2018**, *53*, 175–181. https://doi.org/10.1016/j.nanoen.2018.08.037.
